# Quality of life after intensive care unit: a multicenter cohort study protocol for assessment of long-term outcomes among intensive care survivors in Brazil

**DOI:** 10.5935/0103-507X.20180063

**Published:** 2018

**Authors:** Caroline Cabral Robinson, Regis Goulart Rosa, Renata Kochhann, Daniel Schneider, Daniel Sganzerla, Camila Dietrich, Évelin Carneiro Sanchez, Francine Hoffmann Dutra, Maicon Quadro de Oliveira, Luisa Barbosa Anzolin, Suelen Fardim de Menezes, Rodrigo Jeffman, Denise de Souza, Sâmia Faria da Silva, Luciane Nascimento Cruz, Rodrigo Boldo, Juliana Rezende Cardoso, Daniella Cunha Birriel, Mariana Nunes Gamboa, André Sant'Ana Machado, Juliana Mara Stormosvski de Andrade, Cesar Alencar, Michelle Carneiro Teixeira, Silvia Regina Rios Vieira, Fernanda Caleffe Moreira, Alexandre Amaral, Ana Paula Menezes Silveira, José Mario Meira Teles, Daniela Cunha de Oliveira, Lúcio Couto de Oliveira Júnior, Lívia Correa e Castro, Marli Sarmento da Silva, Rafael Trevizoli Neves, Renata de Andrade Gomes, Cinthia Mucci Ribeiro, Alexandre Biasi Cavalcanti, Roselaine Pinheiro de Oliveira, Juçara Gasparetto Maccari, Paula Pinheiro Berto, Lucieda Araújo Martins, Rui Leandro da Silva Santos, Luciana Yumi Ue, Luciano Serpa Hammes, Tarek Sharshar, Fernando Bozza, Maicon Falavigna, Cassiano Teixeira

**Affiliations:** 1 Escritório de Projetos Programa de Apoio ao Desenvolvimento Institucional/Sistema Único de Saúde (PROADI-SUS), Hospital Moinhos de Vento - Porto Alegre (RS), Brasil.; 2 Centro de Tratamento Intensivo Adulto, Hospital Moinhos de Vento - Porto Alegre (RS), Brasil.; 3 Unidade de Tratamento Intensivo, Hospital Santa Clara, Complexo Hospitalar Santa Casa de Misericórdia de Porto Alegre - Porto Alegre (RS), Brasil.; 4 Unidade de Tratamento Intensivo, Pavilhão Pereira Filho, Complexo Hospitalar Santa Casa de Misericórdia de Porto Alegre - Porto Alegre (RS), Brasil.; 5 Centro de Tratamento Intensivo, Hospital Ernesto Dornelles - Porto Alegre (RS), Brasil.; 6 Centro de Tratamento Intensivo, Hospital Conceição, Grupo Hospitalar Conceição - Porto Alegre (RS), Brasil.; 7 Centro de Tratamento Intensivo, Hospital de Clínicas de Porto Alegre, Universidade Federal do Rio Grande do Sul - Porto Alegre (RS), Brasil.; 8 Unidade de Tratamento Intensivo, Hospital de Urgências de Goiânia - Goiânia (GO), Brasil.; 9 Unidade de Tratamento Intensivo, Hospital Geral Clériston Andrade - Feira de Santana (BA), Brasil.; 10 Unidade de Tratamento Intensivo, Hospital Regional do Baixo Amazonas - Santarém (PA), Brasil.; 11 Unidade de Tratamento Intensivo, HCor-Hospital do Coração, São Paulo (SP), Brasil.; 12 Instituto de Pesquisa, HCor-Hospital do Coração - São Paulo (SP), Brasil.; 13 Coordenação Geral de Atenção Hospitalar, Departamento de Atenção Hospitalar e de Urgência, Secretaria de Atenção à Saúde, Ministério da Saúde - Brasília (DF), Brasil.; 14 Superintendência de Educação, Pesquisa e Responsabilidade Social, Hospital Moinhos de Vento - Porto Alegre (RS), Brasil.; 15 Department of Histopathology and Animal Models, Institute Pasteur - Paris, France.; 16 General Intensive Care, Assistance Publique Hôpitaux de Paris, Raymond Poincaré Hospital, University of Versailles Saint-Quentin en Yvelines - Paris, France.; 17 Instituto D'Or de Pesquisa e Ensino - Rio de Janeiro (RJ), Brasil.

**Keywords:** Critical care outcomes, Quality of life, Cognitive dysfunction, Anxiety, Depression, Stress disorders, Posttraumatic, Disabled persons

## Abstract

**Objective:**

To establish the prevalence of physical, cognitive and psychiatric disabilities, associated factors and their relationship with the qualities of life of intensive care survivors in Brazil.

**Methods:**

A prospective multicenter cohort study is currently being conducted at 10 adult medical-surgical intensive care units representative of the 5 Brazilian geopolitical regions. Patients aged ≥ 18 years who are discharged from the participating intensive care units and stay 72 hours or more in the intensive care unit for medical or emergency surgery admissions or 120 hours or more for elective surgery admissions are consecutively included. Patients are followed up for a period of one year by means of structured telephone interviews conducted at 3, 6 and 12 months after discharge from the intensive care unit. The outcomes are functional dependence, cognitive dysfunction, anxiety and depression symptoms, posttraumatic stress symptoms, health-related quality of life, rehospitalization and long-term mortality.

**Discussion:**

The present study has the potential to contribute to current knowledge of the prevalence and factors associated with postintensive care syndrome among adult intensive care survivors in Brazil. In addition, an association might be established between postintensive care syndrome and health-related quality of life.

## INTRODUCTION

Intensive care units (ICUs) have evolved over time to provide the best human, organizational and technological resources to reduce the mortality of critically ill patients,^(1,2)^ and intensive care development thus centers on the goal of reducing mortality-theoretically the most important outcome.^(3,4)^ However, the increased survival of patients poses new challenges. The reduced mortality of critically ill patients has led healthcare professionals to diagnose and treat a “new disease” caused by complications related to the patient's stay in the ICU.^(2)^

Merely surviving an acute critical illness may not necessarily imply optimal quality of life after discharge. Post intensive care syndrome (PICS) is characterized by physical, cognitive and psychiatric disorders that have the potential to impair the quality of life of patients and often that of their families.^(5-9)^ Complex interactions between comorbidities, complications of the acute critical illness (e.g., hypotension, hypoxia, hypo- or hyperglycemia and polyneuromyopathy), life support (e.g., sedation, mechanical ventilation and dialysis), organizational aspects of intensive care (e.g., restricted contact with family) and adjustment to the post-ICU period (e.g., changes in body image, disabilities, difficulty in returning to work and poor social support network) might impair the functional physical statuses of patients in the long run as well as contribute to the occurrence of cognitive dysfunction, anxiety, depression and posttraumatic stress disorder (PTSD).^(10-15)^

Although the medical literature on PICS is increasing,^(16-23)^ most studies that have been published to date involve specific subpopulations (e.g., patients with sepsis or acute respiratory distress syndrome - ARDS) or assess specific interventions (e.g., mechanical ventilation or dialysis) or isolated complications related to the patient's stay in the ICU (e.g., *delirium*).^(24-27)^ In addition, the evaluations are fragmented (e.g., assessment of cognitive disorders among patients with sepsis or of motor disorders in patients with ARDS) and are not necessarily representative of a large portion of the ICU survivor population. To date, no study has performed a broad-scope, long-term evaluation of the physical, cognitive and mental domains of PICS among a general population of ICU survivors.

The main objective of the present study is to investigate the prevalence of physical disabilities and late cognitive and psychological dysfunctions among ICU survivors in Brazil.

The secondary aims of this study are to perform a long-term evaluation of factors associated with functional dependence, cognitive dysfunction, anxiety, depression and PTSD symptoms among ICU survivors in Brazil and to evaluate the factors associated with readmission and long-term mortality as well as the relationship between physical, cognitive and psychiatric disabilities and health-related quality of life.

## METHODS

**Figure 1:**
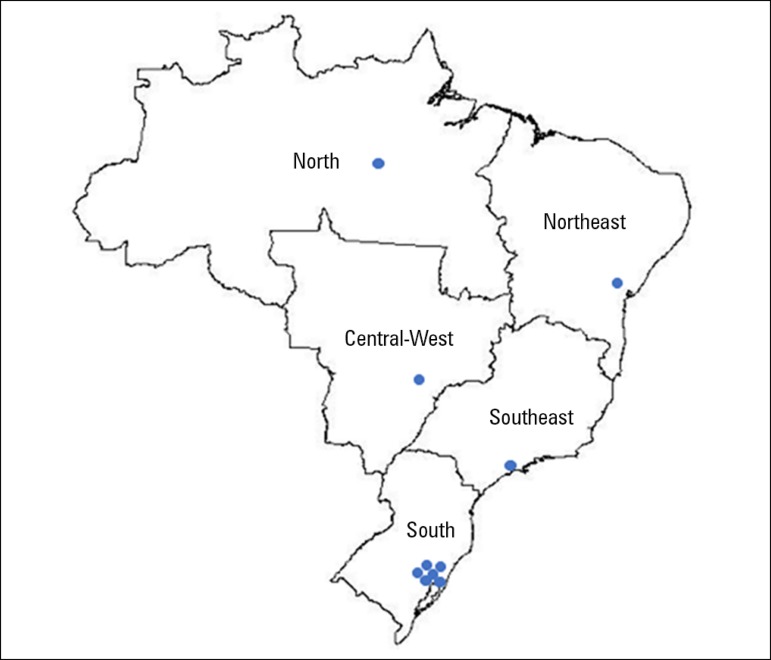
Geographical distribution of participating centers.

**Figure 2:**
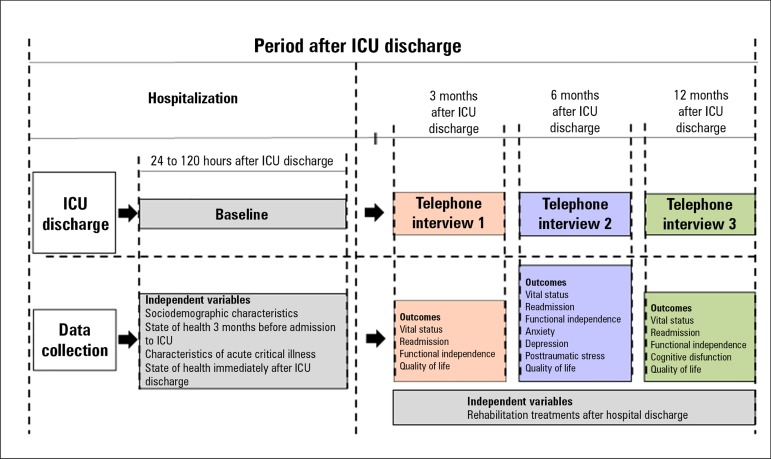
Study design. ICU - intensive care unit.

The present study is a prospective multicenter cohort study. Ten medical-surgical ICUs representative of the 5 geopolitical regions of Brazil (Figure 1) have been selected as study sites. Medical-surgical ICUs in public or private hospitals with 10 or more beds in which the staff manifested interest and had availability to implement the study protocol were selected by convenience sampling. For logistical and financial reasons, 6 of the included ICUs are located in Porto Alegre in the Southern region. To increase the representativeness of the sample, we included one interested hospital with characteristics similar to those of each of the other 4 Brazilian regions. ICU survivors are recruited while still at the hospital, 24 to 120 hours after discharge from the ICU. They are followed up for a period of 12 months by means of structured telephone interviews conducted at 3, 6 and 12 months after ICU discharge (Figure 2).

### Participant eligibility

Patients aged ≥ 18 years who are discharged from the participating ICUs and stay in the ICU ≥ 72 hours (medical or urgent surgery admissions) or ≥ 120 hours (elective surgery admissions) are consecutively included.

The exclusion criteria are as follows: transfer from another ICU or hospital; direct discharge from the ICU to home or direct transfer from the ICU to another hospital; need for respiratory isolation after ICU discharge; impossibility of assessing the patient during the first 5 days after ICU discharge; no available telephone contact; readmission to the ICU; and refusal or withdrawal of agreement to participate.

### Outcomes

#### Functional dependence

The degree of functional dependence will be measured by means of the Brazilian version of the Barthel index (BI)^(28)^ at 3, 6 and 12 months after ICU discharge. The BI measures functional dependence in personal care and mobility. The score ranges from 0 to 100; a higher score indicates less functional dependence.

#### Cognitive dysfunction

Long-term cognitive dysfunction will be investigated by means of the Brazilian version (validated for telephone administration) of the Montreal Cognitive Assessment (tMoCA)^(29)^ at 12 months after the patient's discharge from the ICU. tMoCA scores range from 0 to 22; higher scores indicate better cognitive status.

#### Anxiety and depression symptoms

Anxiety and depression symptoms will be investigated at 6 months after ICU discharge by means of the Brazilian version of the Hospital Anxiety and Depression Scale (HADS).^(30)^ HADS comprises 2 domains, anxiety and depression. The score for each domain ranges from 0 to 21; higher scores indicate greater intensity of anxiety or depression symptoms.

#### Posttraumatic stress

Posttraumatic stress disorder symptoms will be investigated at 6 months after ICU discharge by means of the Brazilian version of the Impact of Event Scale-6 (IES-6).^(31)^ The IES-6 score ranges from 0 to 24; higher scores indicate a greater intensity of PTSD symptoms.

#### Readmission

The rates of unplanned readmission will be calculated at 3, 6 and 12 months after ICU discharge. The outcomes to be analyzed during the 12-month follow-up period are the cumulative incidence of first unplanned readmission after hospital discharge and the number of readmissions per patient.

#### Mortality

The vital statuses of patients will be investigated at 3, 6 and 12 months after ICU discharge.

#### Health-related quality of life

The quality of life of the participants will be investigated by means of the Brazilian version of the Short-Form Health Survey version 2 (SF-12v2).^(32)^ SF-12v2 analyzes health-related quality of life based on the respondent's perception of health aspects in the previous 4 weeks. It comprises 8 domains: general health, physical functioning, physical role function, bodily pain, vitality, emotional role function, mental health and social functioning. The 8 domains are summarized in 2 dimensions, physical and mental. In each case, the score ranges from 0 to 100, and higher scores indicate better health-related quality of life.

#### Other outcomes

The following exploratory outcomes will be considered during follow-up: instrumental activities of daily living, assessed based on the Lawton-Brody index^(33)^ at 12 months after ICU discharge; functional assessment of oral intake, assessed by means of the Functional Oral Intake Scale (FOIS) at 3, 6 and 12 months after ICU discharge;^(34)^ risk of dysphagia, assessed by means of the Eating Assessment Tool-10 (EAT-10)^(35)^ at 3 and 12 months after ICU discharge; level of physical activity, measured using the International Physical Activity Questionnaire (IPAQ)^(36)^ at 3, 6 and 12 months after ICU discharge; return to work (for patients employed at the time of admission to ICU) at 3, 6 and 12 months after ICU discharge; and medical expenses and variation in family income at 3 months after ICU discharge.

Figure 2 shows the time points at which data will be collected during the follow-up period. Trained investigators are responsible for enrolling participants and for collecting baseline data at each participating hospital. Telephone follow-up will be performed from a telephone center located at the study coordination center (*Hospital Moinhos de Vento*). Five sets of independent variables will be analyzed (Table 1) : (1) sociodemographic characteristics; (2) state of health 3 months before admission to the ICU; (3) characteristics of the acute critical illness; (4) state of health immediately after ICU discharge; and (5) rehabilitation treatment received by the patient after hospital discharge. Sociodemographic characteristics, state of health 3 months before admission to the ICU, and characteristics of the acute critical disease will be retrospectively analyzed based on data extracted from medical records and on a structured interview with the participants conducted at 24 to 120 hours after ICU discharge. The patient's state of health immediately after ICU discharge will also be evaluated at that time. Rehabilitation treatments after hospital discharge will be analyzed based on information obtained during the telephone follow-up. All outcomes will be investigated by means of telephone interviews at 3, 6 and 12 months after ICU discharge.

**Table 1:** Independent variables

Sociodemographic characteristics*
Sex; age; ethnicity; marital status; religion; family income; educational level; medical expenses; job regimen; smoking; alcohol consumption; body mass index; Charlson comorbidity index
State of health 3 months before admission to ICU*
Barthel index; Lawton-Brody index; dependence on caregiver; FOIS; home oxygen therapy; noninvasive ventilation at home; physical therapy, psychological care, nutritional care and speech therapy
Characteristics of the acute critical illness*
ICU admission type (clinical, elective surgery, emergency surgery); in-hospital mortality risk at ICU admission (APACHE II or SAPS III); organ dysfunction during stay at ICU (need for mechanical ventilation, vasopressors, dialysis, parenteral nutrition, hemoderivative transfusion, delirium); sepsis or septic shock; acute respiratory distress syndrome; days on mechanical ventilation; need for tracheostomy; ICU-acquired infection (pneumonia, catheter-related bloodstream infection, urinary tract infection)
**State of health immediately after ICU discharge†**
Mini-Mental State Examination; muscle strength on MRC scale; muscle strength on dynamometry; risk of falls according to the Morse Fall Scale; anxiety and depression symptoms on HADS
**Rehabilitation treatments after hospital discharge‡**
Physical therapy, psychological care, nutritional care, speech therapy; need for home care; need for caregiver

ICU - intensive care unit; FOIS - Functional Oral Intake Scale; APACHE II - Acute Physiology and Chronic Health Evaluation II; SAPS III - Simplified Acute Physiology Score III; MRC - Medical Research Council; HADS - Hospital Anxiety and Depression Scale.

* Variables retrospectively analyzed at the time of inclusion in the study;

† Variables analyzed at the time of inclusion in the study;

‡ Variables analyzed by telephone follow-up at 3, 6 and 12 months after intensive care unit discharge.

In cases involving patients who lack the cognitive or physical conditions necessary to provide consent, consent to participation is requested from family members. During the in-person interview at baseline and during the telephone interviews, family members are allowed to answer some objective questions when patients do not exhibit adequate physical or cognitive conditions. Family members are not allowed to respond to the instruments that measure subjective outcomes, such as cognition, anxiety or depression symptoms, PTSD, health-related quality of life, level of physical activity and symptoms of dysphagia.

Despite the use of a protocol to ensure the objectivity of the interviews, spontaneous mentions of clinical complaints are expected to occur during the in-person interview at baseline and during the telephone interviews. Because this is an observational study, no intervention will be performed in such cases. In cases involving medical complaints, the investigators are trained to orient the patient or family members to report the complaint to the assistant physician or to the healthcare staff involved in the patient care. In regard to sensitive questions during interviews at the time of recruitment and during interviews, the investigators will reinforce that participants are entitled to choose not to answer questions that make them uncomfortable.

#### Telephone follow-up

Unlike the baseline assessment performed immediately after ICU discharge, which will be conducted at the participating hospital, telephone follow-up will be performed at a single center. Regardless of the hospital at which the participate receives care, all participants will be followed up by investigators who have been trained in the use of standardized telephone interview methods. To ensure the privacy of the respondents, the telephone interviews will be performed in a secluded room in which only the interviewer is present.

The ICU discharge date serves as a reference for scheduling the telephone interviews. Based on the estimated dates, investigators have a 30-day window period (15 days before and 15 days after the estimated date) to conduct the interviews. Interviews will be rated lost when the participants' telephone lines are disconnected or do not exist or after 10 attempts at different times on several days within the window period. If one interview is lost, contact is nonetheless attempted at the subsequent scheduled interview times.

#### Procedures to ensure the quality of the data

The following procedures will be performed to ensure the quality of the data:

1. To ensure standardization of the study procedures, the investigators responsible for data collection at each participating center will receive training *in loco* prior to the beginning of recruitment.
2. The investigators at each participating center will have access to the study coordination center as a means of dispelling doubts and solving potential problems.
3. The data will be entered on printed standardized data collection forms and stored in an electronic data capture system (REDCap, Vanderbilt University, Nashville, TN, USA). To ensure the adequacy of data transcription, routine double-checking will be performed as data are entered into the electronic data capture system.
4. A data cleaning routine will be applied frequently. The investigators at the participating centers will be contacted in cases of inconsistencies or missing data. This information also provides feedback in regard to the need for retraining.
5. Remote monitoring of data quality will be performed at the study coordination center.
6. Telephone interviews will be taped and audited to verify consistency in data collection. The audio files will be anonymously stored in a server that meets the same security norms as those used for data in electronic medical records. Access to the files, which is restricted to the study team, will require user identification and a password.

The sample size was calculated as the number of participants needed to estimate the prevalence of functional dependence, cognitive dysfunction, anxiety, depression and PTSD symptoms. An adequate sample size for all questions to be answered was chosen. The significance level for estimating the prevalence of each outcome described in table 2,^(12,13,18,19)^ set using the corresponding absolute precision intervals, is 5%. Considering (1) cumulative mortality rates of 15% at 3 months, 25% at 6 months and 40% at one year after ICU discharge,^(1)^ (2) cumulative losses to follow-up of 5% at 3 months, 10% at 6 months and 20% at 12 months, and (3) a potential failure rate of 40% in obtaining responses to telephone follow-up interviews, analysis of 1,212 patients at 3 months, 600 patients at 6 months and 432 patients at 12 months after ICU discharge would require the inclusion of 1,500 participants. The mortality rate at 3 months includes deaths that may occur during the period between transfer from the ICU and hospital discharge. Because this is a multicenter study, we chose to increase the sample size by 10% considering that no estimates of outcome variation exist among the participating centers.

**Table 2:** Sample sizes needed to detect the prevalence of disabilities after discharge from an intensive care unit

Outcome	Time point of assessment (months)	Estimated (%)	Absolute precision (%)	Minimum sample size	Inflated sample size
Functional dependence	3	39^1^	3	1,016	1,118
Posttraumatic stress	6	14^2^	3	545	600
Anxiety	6	33^3^	4	531	585
Depression	6	27^3^	4	474	522
Cognitive dysfunction	12	50^4^	5	385	424

1 According to Dietrich et al.;^(18)^

2 according to Girard et al.;^(13)^

3 according to Myhren et al.;^(12)^

4 according to De Azevedo et al.^(19)^

### Statistical analysis

The analyses to be performed in the present study are aimed at providing a broad view of the physical, cognitive and psychiatric disabilities of ICU survivors in Brazil. Continuous variables will be expressed as the mean and standard deviation or median and interquartile range as appropriate. Categorical variables will be described as absolute and relative frequencies. Regression models will be used to analyze the association between independent variables and outcomes; the distribution of the outcomes of interest probabilities will be fitted using generalized linear models. Graphic analysis will be performed to evaluate the distribution of variables and to verify the assumptions of the regression model. The outcomes relative to physical, cognitive and psychiatric disabilities will be expressed as prevalence rates, mean scores and the corresponding standard deviations. The outcomes measured at each time point, including individual and joint prevalence rates, will also be represented by means of Venn diagrams. Scores on the health-related quality of life questionnaire will be expressed as the mean and standard deviation over time. Mortality after ICU discharge will be represented as survival curves. Functional dependence will also be analyzed as the incidence of BI reduction at each follow-up time point compared to that at baseline. Considering that nonsurvivors and participants unable to respond might represent poorer outcomes, we will perform sensitivity analysis, encompassing the best and worst scenarios, taking into consideration both vital status and unability to respond. A 0.05 significance level will be adopted for all comparisons. R version 3.4.4 software (R-project for statistical computing) will be used for statistical analysis.

The present study will comply with Brazilian National Health Council Resolution no. 466/12. The study protocol was approved by the institutional research ethics committee at the coordination center (CAAE 04258312.4.1001.5330) and by the research ethics committees of all participating centers. The consent form includes information on the study aims, data collection and recording methods and ensures confidentiality and anonymity. Participants are granted the right to withdraw from the study at any time. They are also assured that the collected data will be used only for research purposes.

### Discussion and current status of the study

Health-related quality of life might be defined as the measure of how an individual's normal or expected physical, emotional and social well-being is influenced by a health problem or its treatment.^(37)^ Advances in diagnostic and therapeutic options have resulted in an increased number of patients who survive acute critical illness.^(38)^ Several studies have been performed to analyze short-term mortality within this context. However, research on other relevant aspects, such as prevalence of disabilities after ICU admission, their determinants and their relationship with health-related quality of life, is still insufficient.

After ICU discharge, critically ill patients may develop physical, cognitive and/or psychiatric disorders that lead to prolonged recovery, higher consumption of healthcare resources, and possible impairment of quality of life.^(9,39,40)^ In a systematic review that included 53 studies, ICU survivors consistently reported having a poorer quality of life than healthy controls, even after adjustments were made for age and sex.^(9)^ Despite the plausibility of the association between PICS and impaired health-related quality of life, studies comparing the qualities of life of ICU survivors with and without PICS are scarce.

The strengths of the present study are its prospective multicenter design, its inclusion of a large population of ICU survivors, its use of an *a priori* definition of objective outcomes, and its use of standardized and validated tools for the diagnosis of disabilities after ICU discharge. The limitations of this study derive from the uncertainty regarding the number of patients effectively needed to determine the prevalence of disabilities after ICU discharge, as high rates of mortality and morbidity following critical illness might contribute to losses to follow-up and the inability of the participants to respond to telephone interviews.^(22)^ These aspects might further contribute to underestimation of the prevalence of disabilities after ICU discharge. The use of data reported in studies conducted abroad to calculate the sample size might also interfere with the outcome estimates. Those studies were chosen because they applied the same tools and time points for assessment of each outcome; thus, their design is quite similar to ours.

The study protocol and design were finalized in March 2015. All the investigators from all the participating centers received training on the study procedures *in loco*. We are currently recruiting patients at 10 ICUs representative of the Brazilian geopolitical distribution (Figure 1). As of December 2017, 1,554 patients have been included in the study. We expect that follow-up of all the patients included in the cohort will be completed by December 2018.

## CONCLUSION

The present study will contribute to knowledge regarding the prevalence of disabilities, their determinants and their impact on the qualities of life of intensive care unit survivors in Brazil. The results are expected to give rise to new research questions aimed at investigating the causes of such disabilities and identifying preventive and rehabilitation measures for adult intensive care unit survivors.
